# The effect of intensive resistance exercise and excessive fructose intake on metabolic and physiological responses

**DOI:** 10.1186/s12986-025-00943-y

**Published:** 2025-05-23

**Authors:** Chien-Hua Chen, Shun-Hsi Tsai, Hao-Chien Cheng, Yu-Ting Su, Hung-Wen Liu

**Affiliations:** 1 Department of Physical Education and Sport Sciences, National Taiwan Normal University, 162, Section 1, Heping E. Rd, Taipei City, Taiwan; 2 Office of Physical Education, Tamkang University, New Taipei City, Taiwan; 3 Office of Physical Education, National Taipei University, New Taipei City, Taiwan

**Keywords:** Blood pressure, Kidney function, Uric acid to creatinine ratio, Glutamate pyruvate transaminase

## Abstract

**Background:**

Muscle-derived uric acid (UA) precursors combined with fructose ingestion may increase liver UA production. Temporary hyperuricemia could impact metabolic and physiological responses over a 24-h period. This study examined the effects of intensive resistance exercise (RE) combined with excessive fructose intake on metabolic and physiological responses.

**Methods:**

Twelve healthy young males participated in four trials: RE with fructose intake (EF), RE with water intake (EW), control (no exercise) with fructose intake (CF), and control with water intake (CW). Blood UA, glucose, lipids, blood pressure, and markers of kidney and liver function were measured during fasting and at 0, 0.5, 1, 2, 4, and 24 h before and after exercise.

**Results:**

UA levels in the EF and EW trials were significantly higher than those in the CF and CW trials at all post-exercise time points. The next morning, UA levels in the EF trial remained above 7 mg/dL. Increased glucose levels at 0 and 0.5 h post-exercise and increased creatinine (CRE) levels immediately post-exercise were observed. RE reduced the area under the curve for the estimated glomerular filtration rate (eGFR) and increased systolic blood pressure, mean arterial blood pressure, and the UA/CRE ratio the next morning. Fructose intake increased glutamate pyruvate transaminase (GPT) levels 24 h post-exercise. CRE showed a positive correlation with UA levels, while eGFR was negatively correlated with UA levels in the RE trials. Additionally, GPT levels correlated positively with UA following fructose intake.

**Conclusion:**

Intensive RE combined with excessive fructose intake induced a notable increase in UA levels. This increase in UA levels appeared to be associated with temporary fluctuations in markers related to renal function.

## Background

High intake of fructose-rich industrialized foods is associated with an increased prevalence of obesity, cardiovascular disease, hypertension, and diabetes [24]. Sports and energy drinks are typically marketed as sports performance enhancers and energy store replenishers; however, such drinks usually contain fructose and glucose [27]. Excessive consumption of fructose disrupts the body’s ability to metabolize it [13]. Fructokinase, also called ketohexokinase (KHK), uses adenosine triphosphate (ATP) to phosphorylate fructose into fructose-1-phosphate. In contrast to hexokinases, KHK lacks a negative feedback mechanism for downstream fructose metabolites. Consequently, this rapid phosphorylation of fructose leads to ATP depletion. This reduction in intracellular phosphate levels activates Adenosine monophosphate (AMP) deaminase, which converts AMP into inosine monophosphate (IMP), inosine, and eventually uric acid (UA) [15, 16]. High UA levels impair endothelial function primarily through the reduction of nitric oxide bioavailability in endothelial cells [20] and the promotion of fat accumulation through the stimulation of de novo lipogenesis [19]. These potential detrimental effects may contribute to the development of insulin resistance [19].

Studies have demonstrated that the levels of UA typically increase after resistance exercise (RE) and high-intensity intermittent exercise but not after moderate-intensity continuous exercise [1, 14, 33]. The transient increase in UA levels after intense exercise is primarily attributable to increased UA synthesis engendered by the excessive degradation of adenine nucleotides in skeletal muscles [11, 18]. RE-induced hyperuricemia, in which the UA level exceeds the normal upper limit of 7 mg/dL, has been documented in healthy individuals [1, 33]. The elevations in UA levels can be maintained for hours or even days after RE [33]. Therefore, monitoring and managing the levels of UA are recommended for RE enthusiasts. The combination of muscle-derived UA precursors and fructose ingestion may increase the production of UA in the liver. Moreover, temporary hyperuricemia may affect metabolic and physiological responses, particularly when such responses are evaluated over a 24-h period [19, 20]. However, to our knowledge, no studies have comprehensively explored these assumptions.

To fill the aforementioned research gap, we conducted this study to (1) examine the potential synergistic effect of a single bout of intensive RE combined with excessive fructose intake on metabolic and physiological responses; and (2) explore whether this combination would engender minor adverse effects on blood glucose metabolism, lipid levels, blood pressure levels, and renal and hepatic function. We hypothesized that combining exhaustive RE with fructose intake compared with exercise alone may lead to unfavorable outcomes, including dysglycemia, dyslipidemia, and blood pressure fluctuations.

## Methods

### Participants

Twelve healthy young males were recruited from Taipei City between September 2021 and January 2022. Individuals meeting the following criteria were included: (1) being aged between 20 and 30 years, (2) not having hyperuricemia, (3) not engaging in regular resistance training (less than twice a week), and (4) not having acute or chronic musculoskeletal symptoms or a history of smoking or alcohol or drug abuse. All participants received detailed information regarding the study procedures, potential risks, and objectives. Written informed consent was obtained from all participants. This study was conducted in accordance with the Declaration of Helsinki and was approved by the Research Ethics Committee of National Taiwan Normal University (REC#202012HM020).

### Study design

The participants completed four trials in a randomized order determined through a 4 × 4 Latin square design. These trials involved RE with fructose intake (EF), RE with water intake (EW), control (no exercise) with fructose intake (CF), and control with water intake (CW). A minimum washout period of at least 7 days between sessions was maintained to minimize carryover effects. Block randomization was used to assign the participants to different trials, with each block comprising four participants to ensure a balanced assignment.

Since the interactive effect of RE and fructose has not been previously explored, a formal sample size calculation was not carried out. However, previous studies suggest that a minimum of 10 participants is adequate to observe the impact of RE [8] and fructose intake [30] on UA levels. To account for potential dropouts or missing blood samples, a total of 12 individuals were ultimately included in the study.

### Muscular strength

Muscular strength was evaluated using the one-repetition maximum (1RM) test, which was conducted in the following sequence: deadlift, chest press, squat, and row. Before the test, the participants underwent two familiarization sessions under the guidance of professional trainers to ensure appropriate exercise techniques. The warm-up protocol comprised 5 min of a general warm-up; this was followed by a specific warm-up that involved 10 repetitions at approximately 50% of 1RM and 1 to 2 sets of 3–5 repetitions at approximately 70–80% of 1RM. The test was initiated 2 min after the warm-up, with a progressive increase in load until the participants could no longer complete two repetitions. Rest intervals between attempts lasted 3–5 min, and the final 1RM load was determined within five attempts. This final load was used for subsequent RE sessions. The testing sessions were conducted on 2 days, separated by a minimum of 48 h.

### Experimental trials

The participants were instructed to fast for 12 h and to refrain from any exercise for 48 h before arriving at the laboratory. After 10 min of quiet resting, anthropometric measurements were conducted, and body composition were performed through bioelectrical impedance analysis (InBody720; BioSpace, Seoul, Korea). After fasting blood samples were collected, the participants were instructed to consume a standard breakfast that provided 15% of their total daily caloric intake. This breakfast consisted of a chicken rice ball (carbohydrates: 133.6 kcal, 65%; protein: 23.6 kcal, 11%; and fat: 48.6 kcal, 24%) and unsweetened soy milk (carbohydrates: 8.4 kcal, 17%; protein: 20.4 kcal, 41%; and fat: 23.4 kcal, 47%). After a 1-h break, the participants were instructed to either engage in RE or remain seated for an equivalent duration. During the RE trials, the participants performed a general warm-up, followed by a specific warm-up that involved a set of 10 repetitions at 50% of 1RM. These RE sessions were conducted on a Smith machine (G1-FW161; Matrix Fitness, Johnson Health Tech, Shanghai, China), and they included deadlift, chest press, squat, and row. The training load was set to 70% of 1RM for four sets per exercise. The first three sets comprised eight repetitions, and the fourth set continued until voluntary failure. Movement tempo was maintained at approximately 1:2 s for concentric and eccentric phases. Rest intervals of 90 s were provided between sets, with a 2-min rest period between exercises. All RE trials were supervised by experienced instructors to ensure appropriate form. After the RE sessions or resting sessions in the EF and CF trials, the participants were instructed to consume a 300-mL fructose solution (0.75 g/kg body weight) containing water and high-fructose corn syrup (90%). To avoid discomfort, concentrated lemon juice was added to the fructose solution to reduce its sweetness. In the EW and CW trials, the participants were instructed to consume 300 mL of lemon water. Participants rested in the laboratory for an additional 4 h, during which blood samples were collected immediately and 30 min, 1 h, 2 h, and 4 h after exercise.

### Diet and lifestyle

Throughout the entire experiment, participants maintained their usual daily physical activity. Additionally, for two days before and on the day of each trial, participants refrained from engaging in strenuous exercise and avoided consumption of alcohol, coffee, tea, and sugary drinks. They were also instructed to start water fasting after 8:00 PM on the day before each trial. To mitigate the potential effect of dietary factors on blood UA levels during the experiment, the participants were instructed to refrain from consuming high-purine foods. They were also instructed to document their diet with photographs on the day before and on the day of each trial.

### Blood pressure and heart rate measurement

Blood pressure was measured in a seated position using a blood pressure monitor (HEM-7210,Omron, Kyoto, Japan). Measurements were conducted at least twice at each time point. If the difference between two readings exceeded 10 mmHg, a third measurement was conducted. Mean arterial pressure (MAP) was calculated using the following formula: diastolic blood pressure (DBP) + 1/3([systolic blood pressure, SBP] − DBP) [7].

### Blood sample collection and analysis

A 20-G catheter (Becton Dickinson, San Jose, CA, USA) was inserted into the median cubital vein and connected to a T-shaped extension tube for blood collection. Whole blood samples were collected and used to measure the levels of blood lactate with a lactate analyzer (Lactate Pro 2; Arkray, Kyoto, Japan). These samples were subsequently transferred to heparin-containing centrifuge tubes and centrifuged at 4000 rpm for 10 min at 4 °C. After centrifugation, the plasma supernatant was aliquoted into microtubes and immediately frozen at − 20 °C until further analysis. These plasma samples were analyzed for UA, glutamate oxaloacetate transaminase (GOT), and glutamate pyruvate transaminase (GPT) by using a dry biochemical analyzer (SPOTCHEM EZ SP-4430; Arkray, Kyoto, Japan). In addition, glucose, triglyceride (TG), total cholesterol (TC), high-density lipoprotein cholesterol (HDLc), low-density lipoprotein cholesterol (LDLc), blood urea nitrogen (BUN), and creatinine (CRE) levels were measured using a clinical chemistry analyzer (AU5820; Beckman Coulter, Brea, CA, USA). Estimated glomerular filtration rate (eGFR) was calculated as follows: 175 × CRE^− 1.154^ × age^− 0.203^ [21]. Insulin levels were measured using commercially available enzyme-linked immunosorbent assay kits (Cat. #10-1113-01; Mercodia, Uppsala, Sweden).

### Indices of insulin resistance and β-cell function

Homeostasis model assessment of insulin resistance (HOMA-IR) values were calculated as follows: [fasting insulin (mU/L) × fasting plasma glucose (mg/dL)]/405. Homeostasis model assessment of β-cell function (HOMA-β) values were calculated as follows: [360 × fasting insulin (mU/L)/(fasting glucose (mg/dL) − 63)] [26].

### Statistical analysis

GraphPad Prism 10 (GraphPad Software, La Jolla, CA, USA) was used for statistical analysis. Shapiro–Wilk’s normality test was conducted to evaluate the normality of data distribution. Time course data were analyzed using a three-way repeated-measures analysis of variance (ANOVA; a mixed-effects model was used to account for missing values), with “fructose” (difference between fructose and water intake), “exercise” (difference between RE and rest), and “time” (difference between time points) serving as factors. This analysis yielded various results regarding the significance of independent variables and their interactions with exercise, fructose, time, fructose × time, exercise × time, and fructose × exercise × time (three-way interaction). The trapezoidal rule was used to derive area under the curve (AUC) data at time points spanning from immediately after exercise to 2 h after exercise. The derived AUC data were examined using two-way repeated-measures ANOVA (mixed-effects model to account for missing values), with “fructose” and “exercise” serving as factors. Bonferroni’s post hoc test was conducted to determine between-trial effects at each time point with significant interactions. The relationships between UA levels and biomarkers were assessed using a repeated measures correlation [2, 25]. Significance was set at α = 0.05.

## Results

### Participant characteristics

Table 1 presents the general characteristics of the participants, including their age, height, weight, body mass index, body fat percentage, fructose intake, and UA levels.

**Table 1:** Participant characteristics

Characteristics	*n* = 12
Age (yr)	23 ± 2
Height (cm)	174.6 ± 6.8
Weight (kg)	69.6 ± 7.7
Body mass index (kg/m^2^)	22.8 ± 1.6
Body fat (%)	17.8 ± 5.9
Fructose consumption (g)	52.3 ± 5.8
Uric acid (mg/dL)	5.9 ± 1.3
Note: Values are means ± SD.	

### Uric acid and lactate

**Fig. 1:**
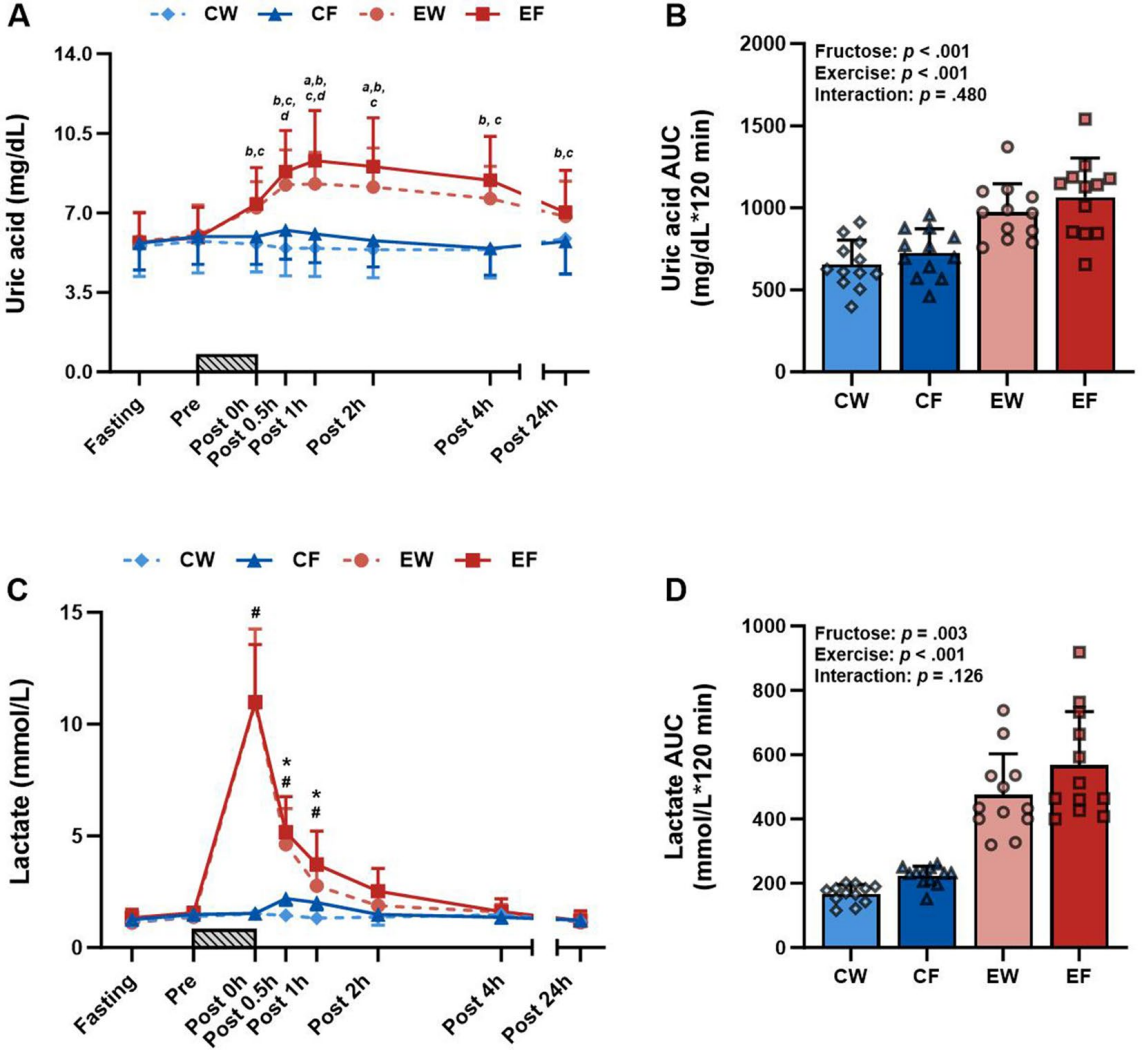
Time course and AUC of uric acid (**A**, **B**) and lactate (**C**, **D**) in the resistance exercise (RE) with fructose intake (EF), RE with water intake (EW), control (no exercise) with fructose intake (CF), and control with water intake (CW) trials (*N* = 12). Time course data were analyzed using a three-way repeated-measures ANOVA (mixed effects model to accommodate missing values) of fructose × time, exercise × time, and fructose × exercise × time. AUC data for the 2-hour period (Post 0-Post 2 h) were analyzed using a two-way repeated-measures ANOVA (mixed effects model to account for missing values) of fructose × exercise, with symbols representing the individual participant values. A gray bar graph indicates the duration of the resistance exercise. Values represent mean ± SD. a: *p* < 0.05 indicates a difference between EF and EW, b: *p* < 0.05 indicates a difference between EF and CF, c: *p* < 0.05 indicates a difference between EW and CW, d: *p* < 0.05 indicates a difference between CF and CW, #: *p* < 0.05 indicates a difference between resistance exercise trial and sedentary rest trial, *: *p* < 0.05 indicates a difference between the fructose intake trial and the water intake trial

The UA levels exhibited a three-way interaction (Fig. 1A). UA levels were consistently higher in the EF and EW trials than in the CF and CW trials at all post-exercise time points. Specifically, UA levels were significantly higher in the EF trial than in the EW trial at 1 and 2 h after exercise. Similarly, UA levels were significantly higher in the CF trial than in the CW trial at 0.5 and 1 h after exercise. No significant two-way interaction was observed in the AUC data obtained for UA (Fig. 1B). By contrast, significant main effects were observed in the AUC values derived for both fructose intake and exercise.

Lactate responses exhibited an exercise × time interaction, indicating higher levels of lactate at 0, 0.5, and 1 h after exercise in the exercise trials than in the rest trials (Fig. 1C). A fructose × time interaction was also observed, with high lactate levels observed at 0.5 and 1 h after exercise in the fructose trials. No significant two-way interaction was observed in the AUC values derived for lactate (Fig. 1D). By contrast, significant main effects were observed in the AUC values derived for lactate for both fructose intake and exercise trials.

### Glucose and lipid profiles

**Fig. 2:**
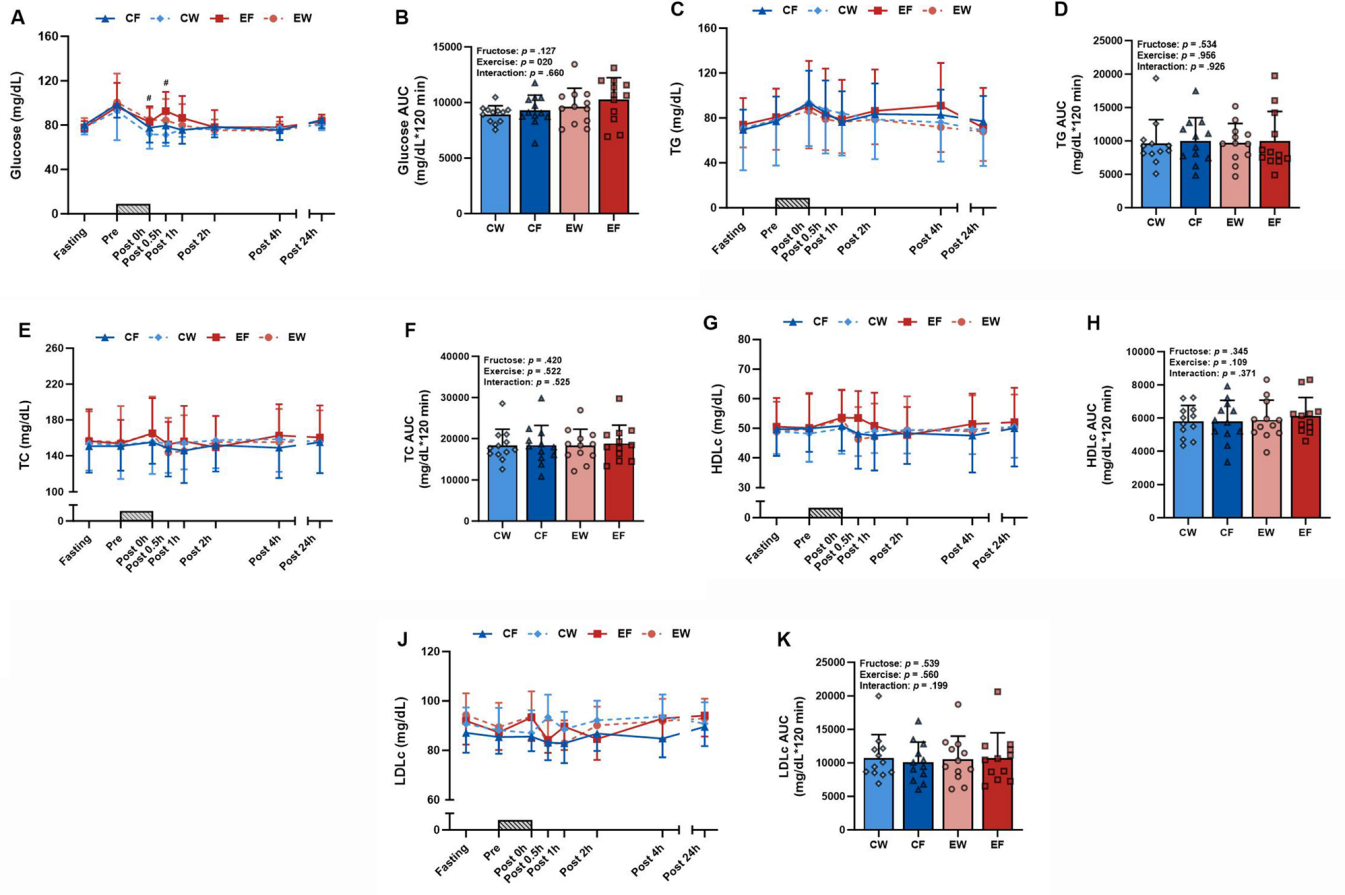
Time course and AUC of glucose (**A**, **B**), TG (**C**, **D**), TC (**E**, **F**), HDLc (**G**, **H**), and LDLc (**J**, **K**) in the resistance exercise (RE) with fructose intake (EF), RE with water intake (EW), control (no exercise) with fructose intake (CF), and control with water intake (CW) trials (*N* = 12). Time course data were analyzed using a linear mixed model, examining three-way interactions of fructose × time, exercise × time, and fructose × exercise × time. AUC data for the 2-hour period (Post 0-Post 2 h) were analyzed using a linear mixed model, focusing on two-way interactions of fructose × exercise, with symbols representing the individual participant values. A gray bar graph indicates the duration of the resistance exercise. Values represent mean ± SD. #: *p* < 0.05 indicates a difference between the resistance exercise trial and the sedentary rest trial

A significant exercise × time interaction was observed for glucose responses, indicating higher levels of glucose in the exercise trials at 0 and 0.5 h after exercise than in the rest trials (Fig. 2A). A significant main effect was also observed in the AUC values for glucose in the exercise trials (Fig. 2B). By contrast, no difference was observed in insulin, HOMA-IR, or HOMA-β between the four trials during fasting and at 24 h after exercise (Table 2). In addition, no three-way interactions were observed in TG, TC, HDLc, or LDLc (Fig. 2C, E, G, and J). Similarly, no two-way interactions or main effects were observed in the AUC values for TG, TC, HDLc, or LDLc (Fig. 2D, F, H, and K).

**Table 2:** Fasting levels of response parameters

	CW		CF		EW		EF		TIME x EX x FRU	TIME x EX	TIME x FRU
Variables	Fasting	Post 24 h	Fasting	Post 24 h	Fasting	Post 24 h	Fasting	Post 24 h			
Insulin (mU/L)	4.4 ± 1.7	3.95 ± 1.33	3.99 ± 1.53	4.85 ± 2.7	4.11 ± 3.05	3.54 ± 1.64	4.52 ± 2.2	3.97 ± 2.11	0.316	0.293	0.363
HOMA IR	0.85 ± 0.36	0.78 ± 0.26	0.8 ± 0.37	1.01 ± 0.53	0.83 ± 0.69	0.72 ± 0.34	0.87 ± 0.44	0.83 ± 0.46	0.386	0.318	0.252
HOMA beta	102.52 ± 38.55	89.96 ± 36.59	90.96 ± 33.29	94.82 ± 86.19	94.28 ± 41.92	75.24 ± 38.44	106.08 ± 51.71	72.68 ± 37.68	0.371	0.180	0.953
UA/CRE ratio	6.58 ± 1.68	6.71 ± 2.11	6.56 ± 1.33	6.51 ± 1.63	7.18 ± 1.89	8.08 ± 2.2 ^ab^	6.47 ± 1.31	8.01 ± 2.03 ^ab^	0.087	0.002*	0.295
GOT (mg/dL)	15.42 ± 3.8	16.17 ± 2.95	14.83 ± 2.82	15 ± 3.25	18.67 ± 9.74	15.75 ± 3.67	16.83 ± 4.8	18.75 ± 6.52	0.154	0.588	0.242
GPT (mg/dL)	13.67 ± 5.97	14 ± 5.82	12.67 ± 3.28	14 ± 5.56^a^	14.42 ± 4.96	14 ± 4.69	13.17 ± 3.69	15 ± 4.13^a^	0.276	0.832	0.0164*

**Fig. 3:**
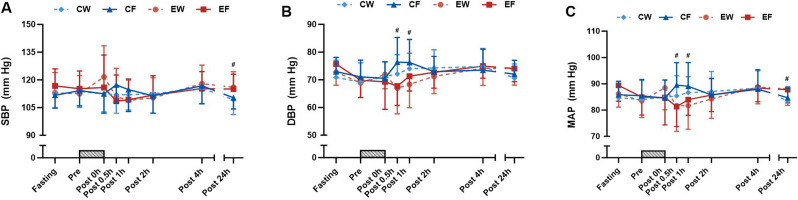
Time course of SBP (**A**), DBP (**B**), and MAP (**C**) in the resistance exercise (RE) with fructose intake (EF), RE with water intake (EW), control (no exercise) with fructose intake (CF), and control with water intake (CW) trials (*N* = 12). Time course data were analysed using a linear mixed model, examining three-way interactions of fructose × time, exercise × time, and fructose × exercise × time. A gray bar graph indicates the duration of the resistance exercise. Values represent mean ± SD. #: *p* < 0.05 indicates a difference between the resistance exercise trial and the sedentary rest trial

Significant exercise × time interactions were observed for SBP and MAP responses, indicating higher SBP and MAP levels in the exercise trials at 24 h after exercise than in the rest trials (Fig. 3A and C). The levels of DBP and MAP at 0.5 and 1 h after exercise were lower than those in the rest trials (Fig. 3B and C).

### Kidney and liver markers

**Fig. 4:**
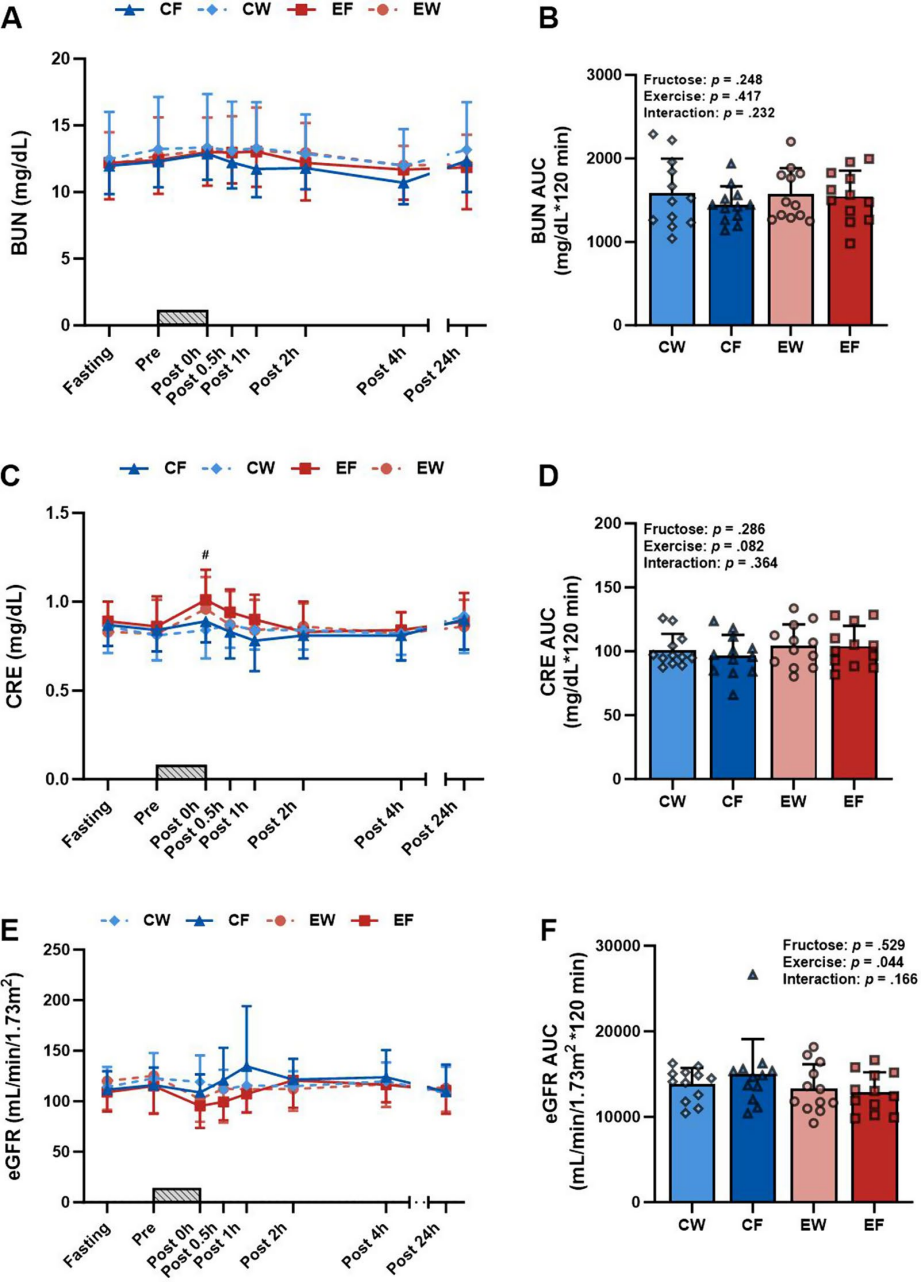
Time course and AUC of BUN (**A**, **B**), CRE (**C**, **D**), and eGFR (**E**, **F**) in the resistance exercise (RE) with fructose intake (EF), RE with water intake (EW), control (no exercise) with fructose intake (CF), and control with water intake (CW) trials (*N* = 12). Time course data were analyzed using a linear mixed model, examining three-way interactions of fructose × time, exercise × time, and fructose × exercise × time. AUC data for the 2-hour period (Post 0-Post 2 h) were analyzed using a linear mixed model, focusing on two-way interactions of fructose × exercise, with symbols representing the individual participant values. A gray bar graph indicates the duration of the resistance exercise. Values represent mean ± SD. #: *p* < 0.05 indicates a difference between the resistance exercise trial and the sedentary rest trial

The levels of CRE were higher in the exercise trials than in the rest trials immediately after exercise (Fig. 4C). No three-way interactions were observed for BUN or eGFR (Fig. 4A and E). Similarly, no two-way interactions were observed for the AUC value for BUN and CRE (Fig. 4B and D). The AUC value for eGFR was higher in the exercise trials than in the rest trials (Fig. 4F). An exercise × time interaction was observed, with the exercise trials exhibiting a greater UA/CRE ratio at 24 h after exercise when compared with the fasting and rest trials (Table 2). A fructose × time interaction was also observed, with fructose intake resulting in higher GPT levels at 24 h after exercise when compared with the water intake trials (Table 2). A repeated measures correlation was conducted to further explore the relationship between UA levels and other biomarkers [2, 25]. CRE levels and eGFR were correlated with UA levels following RE, respectively (*r*_rm_ = 0.17, 95% CI = 0.021 to 0.305, *p* = 0.025; *r*_rm_ = -0.18, 95% CI = -0.32 to -0.038, *p* = 0.014). GPT was correlated with UA levels following fructose intake (*r*_rm_ = 0.62, 95% CI = 0.365 to 0.784, *p* < 0.001).

## Discussion

This study examined the metabolic and physiological effects of intensive RE and excessive fructose intake (0.75 g/kg body weight) in healthy young males. We discovered that intensive RE combined with excessive fructose intake resulted in higher UA levels than did exercise alone, with hyperuricemia (exceeding 7 mg/dL) persisting overnight following exercise independent of fructose intake. On the next morning, we observed elevated blood pressure levels and high UA/CRE ratios, along with increased GPT levels after fructose intake. These findings indicate the potential metabolic and clinical effects of exercise-induced hyperuricemia and fructose consumption; hence, the long-term effects of exercise-induced hyperuricemia and fructose consumption on health outcomes warrant further investigation.

UA is the endpoint metabolite of purine metabolism, and it typically increases in circulation after intense exercise [18]. During RE, contracting muscles primarily rely on ATP–PC and anaerobic systems, resulting in increased adenine nucleotide degradation [3]. Adenylate kinase catalyzes the conversion of two adenosine diphosphate molecules into one ATP molecule and one AMP molecule, facilitating the ATP replenishment process. Subsequently, the AMP molecule is irreversibly degraded by AMP deaminase, resulting in the production of IMP and ammonia. The IMP is converted into hypoxanthine, which then enters the bloodstream from muscle tissues. Finally, this metabolic pathway leads to the synthesis of UA in the liver [11, 18]. Our findings are consistent with those of previous studies, indicating that RE induces hyperuricemia in healthy individuals [1, 29, 33]. In addition to increasing the production of UA, temporary hyperlactatemia induced by intensive RE may diminish the excretion of UA through urate transporter 1 [10, 35].

Individuals with hyperuricemia may experience certain health problems resulting from spikes in blood UA levels after intensive RE. In this study, we observed that RE resulted in higher UA levels when it was combined with fructose intake than when it was performed RE alone, with the peak difference reaching approximately 1 mg/dL at 1 h after exercise. Sports and energy drinks containing fructose are often marketed for individuals seeking to enhance their exercise performance or energy levels [27]. Our findings demonstrate that even exercise performed with excessive fructose intake (52.3 ± 5.8 g), which exceeds the American Heart Association recommends < 36 g of added sugar per day for males, considerably increased the levels of UA when compared with exercise performed without fructose. In this study, we observed that hyperuricemia persisted overnight in the EF trial, whereas the levels of UA in the EW trial (6.84 mg/dL) approached the upper normal limit. Persistent hyperuricemia may lead to the deposition of monosodium urate crystals, thereby increasing the risk of gout [28]. Therefore, long-term monitoring of the effects of exercise-induced hyperuricemia on metabolic and physiological responses in fitness enthusiasts and athletes is essential.

Our study revealed a significant increase in glucose levels after exhaustive RE. Elevated blood glucose levels after RE can be attributed to increased liver glycogenolysis stimulated by stress hormones, such as epinephrine and cortisol [32]. Temporary hyperuricemia induced by exhaustive RE and fructose intake appeared to have marginal effects on insulin sensitivity and secretion, as demonstrated by the derived HOMA-IR and HOMA-β values in the participants. Nevertheless, in individuals with prediabetes and diabetes, high-intensity or high-volume RE may not be the most suitable exercise regimen for controlling blood glucose. Notably, prolonged excessive intake of fructose may lead to obesity, high blood lipid levels, and other metabolic disorders [31].

In line with our study, previous research reported that blood pressure temporarily decreased after exercise [4]. Furthermore, our study observed increased blood pressure on the next morning after exhaustive RE when compared with the control. A possible explanation for this is that elevated UA levels could result in endothelial dysfunction, reduce the availability of nitric oxide, and lead to vasoconstriction, thereby increasing blood pressure [20]. High levels of UA can also activate the renin–angiotensin system, further increasing blood pressure [6]. However, increased blood pressure may be due to other biological processes in the post-exercise state. Therefore, monitoring and managing the levels of UA are essential for individuals with hypertension, particularly those who engage in regular high-intensity or high-volume resistance training.

CRE is a primary marker of renal function [17]. In this study, we observed a marked increase in CRE levels immediately after RE. These patterns can be attributed to the exercise-induced activation of the sympathetic system, which leads to the vasoconstriction of afferent arterioles, thereby reducing renal blood flow and subsequently lowering the eGFR [9]. In our healthy participants, high CRE levels induced by exhaustive RE gradually decreased after fluid consumption. Training to failure also increased the UA/CRE ratio on the next morning. The UA/CRE ratio is widely used as a biomarker for evaluating the risks of metabolic syndrome, hypertension, and cardiovascular disease [5, 22, 34]. This clinical index should be interpreted with caution, particularly in athletes and fitness enthusiasts who frequently engage in high-intensity exercise. Additionally, after excessive fructose intake, GPT levels elevated the next morning. We further conducted a repeated measures correlation to explore the relationship between UA levels and these biomarkers. In the RE trials, CRE displayed a positive correlation with UA levels, while eGFR showed a negative correlation with UA levels. GPT exhibited a positive correlation with UA levels following fructose intake. These findings suggest that elevated UA levels are associated with temporary impairments in renal and liver functions.

This study has some limitations. First, although studies are increasingly examining sex-based disparities in metabolic and physiological responses, our study exclusively focused on young males because of the higher prevalence of hyperuricemia among males than among females [12]. Considering the inverse relationship between hyperuricemia and estrogen levels, future studies should particularly explore the effects of intensive RE on postmenopausal females [23]. Second, this study included healthy, untrained young adults who were at a low risk of metabolic syndrome. Responses to RE and fructose consumption may differ in trained individuals, and this should be considered in future research. Individuals with hyperuricemia, hypertension, and prediabetes should be particularly aware of the effects of intensive RE and fructose consumption on them. Regular monitoring of UA levels after exercise is also essential for these high-risk groups. Further research is warranted to determine the long-term effects of hyperuricemia induced by exhaustive RE in conjunction with fructose intake, particularly on the development of metabolic syndrome.

In conclusion, intensive RE combined with excessive fructose intake resulted in higher UA levels than exercise alone, with hyperuricemia persisting until the next morning, regardless of fructose intake. The following morning, elevated blood pressure, increased UA/CRE ratios, and higher GPT levels were observed after fructose intake. The increase in UA levels was associated with transient changes in renal function markers. These results highlight the potential metabolic and clinical implications of exercise-induced hyperuricemia and fructose intake, warranting further investigation into their long-term health effects.

## Data Availability

No datasets were generated or analysed during the current study.

## Abbreviations

AMP: Adenosine monophosphate
ANOVA: Analysis of variance
ATP: Adenosine triphosphate
AUC: Area under the curve
BUN: Blood urea nitrogen
CRE: Creatinine
eGFR: Estimated glomerular filtration rate
GOT: Glutamate oxaloacetate transaminase
GPT: Glutamate pyruvate transaminase
HDLc: High-density lipoprotein cholesterol
HOMA-β: Homeostasis model assessment of β-cell function
HOMA-IR: Homeostasis model assessment of insulin resistance
IMP: Inosine monophosphate
KHK: Ketohexokinase
LDLc: Low-density lipoprotein cholesterol
MAP: Mean arterial pressure
1RM: One-repetition maximum
RE: Resistance exercise
SBP: Systolic blood pressure
TC: Total cholesterol
TG: Triglyceride
UA: Uric acid
